# Commercial Mannoproteins Improve the Mouthfeel and Colour of Wines Obtained by Excessive Tannin Extraction

**DOI:** 10.3390/molecules26144133

**Published:** 2021-07-07

**Authors:** Alessandra Rinaldi, Alliette Gonzalez, Luigi Moio, Angelita Gambuti

**Affiliations:** 1 Dipartimento di Agraria, Sezione di Scienze della Vigna e del Vino, Università degli Studi di Napoli Federico II, Viale Italia, Angolo Via Perrottelli, 83100 Avellino, Italy; a.gonzalessifuentes@studenti.unina.it (A.G.); moio@unina.it (L.M.); angelita.gambuti@unina.it (A.G.); 2 Biolaffort, 126 Quai de la Souys, 33100 Bordeaux, France

**Keywords:** mannoproteins, mouthfeel, astringency, subquality, colour, pressing, extended maceration, Sangiovese

## Abstract

In the production of red wines, the pressing of marcs and extended maceration techniques can increase the extraction of phenolic compounds, often imparting high bitterness and astringency to finished wines. Among various oenological products, mannoproteins have been shown to improve the mouthfeel of red wines. In this work, extended maceration (E), marc-pressed (P), and free-run (F) Sangiovese wines were aged for six months in contact with three different commercial mannoprotein-rich yeast extracts (MP, MS, and MF) at a concentration of 20 g/hL. Phenolic compounds were measured in treated and control wines, and sensory characteristics related to the astringency, aroma, and colour of the wines were studied. A multivariate analysis revealed that mannoproteins had a different effect depending on the anthocyanin/tannin (A/T) ratio of the wine. When tannins are strongly present (extended maceration wines with A/T = 0.2), the MP conferred mouthcoating and soft and velvety sensations, as well as colour stability to the wine. At A/T = 0.3, as in marc-pressed wines, both MF and MP improved the mouthfeel and colour of Sangiovese. However, in free-run wine, where the A/T ratio is 0.5, the formation of polymeric pigments was allowed by all treatments and correlated with silk, velvet, and mouthcoat subqualities. A decrease in bitterness was also obtained. Commercial mannoproteins may represent a way to improve the mouthfeel and colour of very tannic wines.

## 1. Introduction

The key step in the production of red wine is the maceration of the solid parts of the berries during fermentation. In this step, important phenomena occur in which the phenolic compounds of the grapes are involved: the release of part of them from the skins and seeds into the must, reactions among themselves and with other metabolites of fermentation, and the absorption on grape pomaces and yeast lees [1]. The phenolic compounds constitute a wide group of compounds, and, among them, the most important in winemaking are the anthocyanins extractable from the skins and the proanthocyanidins (namely condensed tannins) extractable from the skins and seeds [2]. Grape maceration is a critical point in red wine production, as an excessive extraction of tannins and/or low extraction of anthocyanins or loss of part of them during the process can determine an unbalanced ratio between these classes of phenolic compounds. This may cause defects such as astringency and bitterness, which reduce the commercial value of wines [3]. Apart from the specific varietal composition in anthocyanins and tannins of the berry, numerous factors can modulate the extraction of these two important groups of compounds during the initial stages of winemaking [4,5]. One of the technological practices that often determine an excessive extraction of tannins is the prolonged maceration after the end of alcoholic fermentation [6]. This practice is usually applied to obtain wines richer in phenolic compounds and with a longer shelf-life, but sometimes, these wines are too rich in phenolic compounds responsible for bitterness and astringency [7]. Fining treatments with high doses of animal and vegetable proteins are necessary to diminish the content of flavanols and proanthocyanidins and decrease the undesired mouthfeel sensations elicited by these compounds [8,9]. Fining practices, on the other hand, can impoverish the aroma of wines, so the commercial value of these products may be low anyway. Unbalanced red wines are also produced by the marc-pressing of wines shortly after the end of maceration. Usually, the free-run juice is used to produce higher quality wines that are richer in compounds easily extracted from the grape skin and seeds such as anthocyanins and lower molecular weight tannins, which are characterised by more pleasant mouthfeel sensations [10]. The corresponding marc-pressed wines are lower quality wines, because they are richer in bitter and astringent compounds, such as flavanols and proanthocyanidins, which are extracted from the skin and seeds during the pressing of the marcs [11]. However, sometimes, these marc-pressed wines can be rich in aromatic compounds, and with appropriate treatment, they could have a higher commercial value [12,13].

In addition to fining agents capable of precipitating phenolic compounds such as albumin, gelatin, and some vegetable proteins, which can be used to improve the mouthfeel properties of wines too rich in tannins, mannoproteins can also be used to improve the in-mouth characteristics of red wines [14].

Mannoproteins represent major polysaccharides found in wine, because they are released from the yeast cell wall during alcoholic fermentation and wine ageing [15,16]. In recent decades, several commercial mannoproteins have been added to wine, because they confer favourable oenological properties, such as the decrease of astringency, the improvement of mouthfeel sensation [14,17], the increase of colour [17], and protein and tartrate stability [18].

Mannoproteins or yeast products rich in mannoproteins are used for various types of wines, such as still white wine [19] and red wine [17], as well as white and rosé sparkling wines [20]. However, they are rarely used to treat wines that are very rich in astringent and bitter tannins, as those obtained by excessive extractive procedures like prolonged maceration and marc-pressing.

The effectiveness of commercial mannoproteins on protein stabilisation, phenolic compounds, and the chromatic and sensory properties of wine depend on the structural characteristics of mannoproteins [19]. Recently, Manjon et al. [21] also showed that the formation of salivary protein–mannoprotein systems, mainly involved in altering astringency sensations, depends on the structural characteristics and hydrophobicity of mannoproteins. Therefore, the possible use of commercial mannoproteins to modulate the astringency and bitterness attributes of tannin-rich red wines should consider different preparations. On the other hand, the contact time between mannoproteins and red wine is important to reach the colloidal state able to induce a significant variation in the perceived sensations [17]. In this study, three commercial mannoproteins were tested to remediate the excessive astringency and bitterness of red wines produced by prolonged maceration and marc-pressing, considering the contemporary effect on chromatic wine characteristics and aroma compounds.

## 2. Results

### 2.1. The Content in BSA-Reactive Tannins and Vanillin-Reactive Flavans

The BSA-reactive tannins and vanillin-reactive flavans in extended maceration (E), marc-pressed (P), and free-run (F) Sangiovese wines were measured before (t0) and after the ageing on mannoproteins for six months.

**Figure 1:**
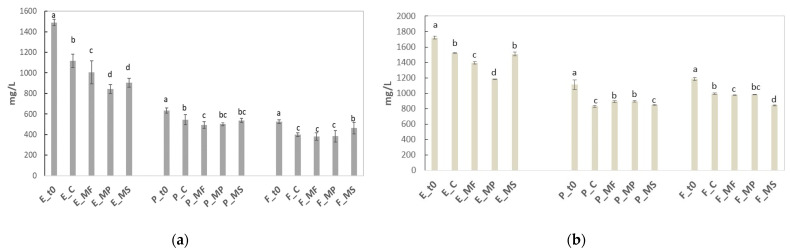
BSA-reactive tannins (**a**) and vanillin-reactive flavans (**b**) after six months of ageing on mannoproteins at 20 g/hL in extended maceration (E), marc-pressed (P), and free-run (F) Sangiovese wines. C represents the control wine; MF, MP, and MS are the mannoprotein treatments. t0 represents the wine before ageing. According to Fisher’s LSD analysis, histograms with different letters indicate significant differences for each wine typology (*p* < 0.05).

In Figure 1a, the prolonged contact between grape solids and wine during the extended maceration process extracted around 1500 mg/L of BSA-reactive tannins (E-t0), compared to 600 mg/L of marc-pressed wine (P-t0) and 500 mg/L of free-run (F-t0) wine. After six months, the control wines E-C, P-C, and F-C showed a significantly lower concentrations of proanthocyanidins with respect to t0. In E wines, the treatments with MP and MS induced a greater reduction in BSA-reactive tannins; in P wines, only MF was efficient in reducing these compounds; in F wines, there were no differences between the control and treated wines, except for MS, which showed a higher tannin concentration than the control but a lower one than before ageing.

Extended maceration also resulted in a high extraction of vanillin-reactive flavans (Figure 1b). The content in E-t0 was around 1700 mg/L compared to 1200 mg/L in P-t0 and F-t0 wines. The vanillin-reactive flavans in the control wines decreased concerning the t0 in all the wines after six months. The decrease was also observed after the treatments with MF and MP in E wines and MF and MS in F wines, with MS being the most effective. In contrast, in the P-MF and P-MP wines, the contents of flavans were higher than in the control, probably due to reduced precipitation over time.

### 2.2. The Effect of Mannoproteins on the Colour of Wines

We evaluated the impact of mannoproteins after ageing on colour by measuring the colour intensity; hue; total anthocyanins; polymeric pigments content; and CIELab coordinates (L*, a*, b*, and ΔE), as shown in Table 1.

In E wines, the total anthocyanin content decreased after ageing on mannoproteins, mainly due to the MP treatment. Colour intensity and redness (a*) were also reduced in E-MP wine. However, a higher amount of polymeric pigments was formed, indicating that wine still had a red colour with violet hues, as shown by the b* coordinate (yellow–blue). The lightness (L*) increased in all treatments, indicating a more vivid colour. The ΔE represents the difference in colour between the control and treated wines, and the value 5.34 of E-MP showed that this wine had a different colour, easily detectable by the human eye. In Table 1, the decrease of total anthocyanins in P-MS and P-MP was lower than in P-C. Moreover, the P-MS showed a higher colour intensity and hue. Polymeric pigments were mainly formed in P-MF, although no differences were observed with the control in a* and b*. The P-MP was the wine with a colour difference (ΔE = 2.98) detectable to untrained eyes, probably due to the lower lightness and higher blue nuances than the other wines. A different effect of mannoproteins can be observed in free-run wines on the colour parameters.The total anthocyanins did not differ significantly in free-run wines (F), while the colour intensity was higher in F-MF and lower in F-MP than in F-C. A slight increase in hue was observed in F-MS and F-MF. Yet, the polymeric pigments were significantly increased after the treatment with all mannoproteins in free-run wines. In F-MF, a high redness was also observed. However, no evident colour differences were denoted in the treated wines.

### 2.3. The Effect of Mannoproteins on the Mouthfeel of Wines

**Figure 2:**
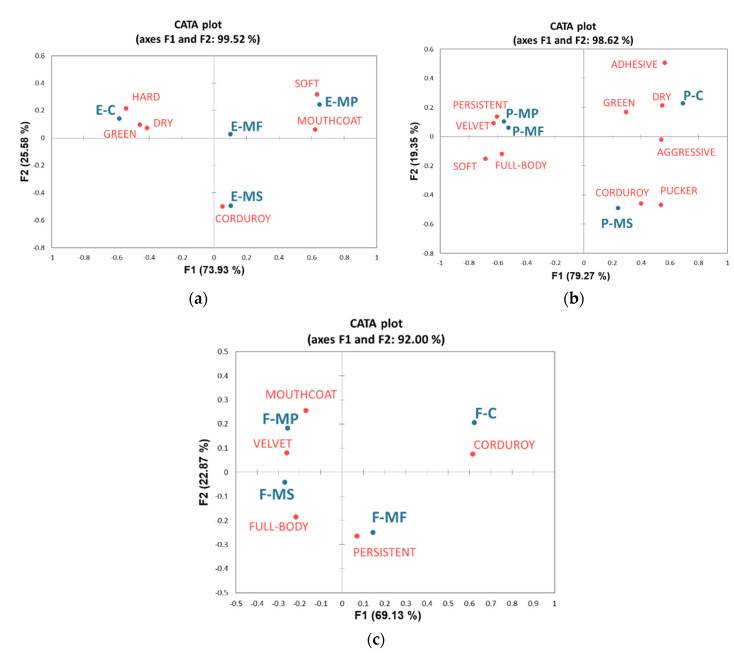
The mouthfeel profile of Sangiovese wines after six months of ageing with mannoproteins by the CATA analysis. The significant terms (*p* < 0.01) were plotted for each wine typology: (**a**) extended maceration (E), (**b**) marc-pressed (P), and (**c**) free-run (F) wines. C represents the control wine; MF, MP, and MS are the mannoprotein treatments at 20 g/hL.

After six months of ageing on mannoproteins, the mouthfeel profile of Sangiovese wines was evaluated using 16 attributes of astringency (Supplementary Table S1), which were analysed by the CATA analysis. The significant terms (*p* < 0.01) were plotted for each wine typology in Figure 2. Figure 2a shows the subqualities of the wines obtained by extended maceration (E-C) and treated with mannoproteins (E-MS, E-MP, and E-MF).

The explained inertia was 99.52% by the first two coordinates and permitted the separation of the samples according to their astringency attributes. E-C was characterised principally by dry, hard, green terms, i.e., high astringency felt with bitterness and acidity. The primary sensation of E-MS was corduroy, a feeling of a slight wrinkling of the soft palate that can be felt by tongue movements. E-MF was instead very similar to the other wines and did not differ from either the control or treated wines. E-MP wine resulted in soft and mouthcoating sensations, indicating that the MP represented the most suitable treatment to improve the mouthfeel of extended maceration wine. E-MP was also the wine with the lowest content of BSA-reactive tannins and vanillin-reactive flavans.

Figure 2b showed the CATA plot of the marc-pressed wines (P) after six months of ageing on the MS, MF, and MP mannoproteins. The first and second dimensions explained 98.62% of the total inertia and allowed a clear separation between the P-C and P-MS from the P-MP and P-MF. Green, dry, adhesive, and aggressive sensations characterised the control P-C, and the P-MS differed from the latter for the corduroy and pucker terms. In contrast, the treatment with MF and MP mannoproteins conferred positive subqualities to the wines: velvet, soft, full-body, and persistent. For the marc-pressed wines, the most evident effect on mouthfeel was similarly obtained with the MP and MF mannoproteins, although the BSA-reactive tannins and vanillin-reactive flavans did not show noticeable variations after these treatments.

Figure 2c showed the mouthfeel profile of Sangiovese free-run wines after six months of ageing on mannoproteins using the CATA analysis. The corduroy term highly characterised F-C. Even if each wine was different from the control, the treated wines were similar in their mouthfeel profiles. In particular, F-MF was persistent, indicating that the overall sensation associated with the aftertaste lasted long in the mouth. F-MP was principally velvety and mouthcoating, while F-MS was perceived as full-bodied.

### 2.4. Relationships between Subqualities, Colour Parameters, and Phenolic Content of Wines

It is essential to consider how the colour evolves together with the variation in mouthfeel during ageing with mannoproteins. For this reason, relationships between different variables such as the astringency subqualities (silk, velvet, dry, corduroy, adhesive, aggressive, hard, soft, mouthcoat, rich, full-body, green, grainy, satin, pucker, and persistent); colour parameters (colour intensity, hue, a*, b*, L*, and polymeric pigments); and phenolic content of the wine (total anthocyanins, BSA-reactive tannins, and vanillin-reactive flavans) were studied. A Multiple Factor Analysis (MFA) was carried out on each wine typology to characterise and find relationships between the variables and factors, as shown in Table 2.

For extended maceration wines (E), the first two dimensions (F1 and F2) of the MFA accounted for 93.4% of the variance of the experimental data, representing 75.9% and 17.5% of the variance, respectively. The F1 and F2 of the MFA for marc-pressed wines (P) accumulated 61.2% and 35% and, for free-run wines (F), 50.8% and 34.2%, totalling 96.3% and 85% of the initial variability, respectively. The eigenvalue of the first dimension indicated that it could be considered a significant direction in explaining the dispersion of analytical parameters (colour and phenolic content) and the frequency table of the CATA terms (astringency subqualities), being E > P > F. The first dimension of the MFA of E was positively correlated with lightness (L*) and hue and with the subqualities velvet, soft, mouthcoat, satin, and persistent. The factor score related to the treatment MP was positively loaded on F1, indicating that the wine showed positive subqualities, a more vivid colour, and a higher hue than other wines. On the same factor F1, the control wine was negatively projected, characterised by dry, adhesive, hard, aggressive, green, grainy, and pucker terms, which are correlated with the content BSA-reactive tannins, total anthocyanins, flavans, colour intensity, redness (a*), and yellowness–blueness direction (b*). The colour and the phenolic content similarly contributed to F1 (33.7%). The factor F2 was formed by the contribution of the variables subquality and colour by 55.2% and 30.1%. The highest factor score of MS was loaded on F2, indicating an association with polymeric pigments (of the variable colour) and silk, full-body, and corduroy (of the variable subqualities). This suggests that the MS treatment can impart colour stability and interesting mouthfeel characteristics to extended maceration wine. MF, on the other hand, was associated with F3 and aromatic richness (rich); however, it did not differ from the other wines.

For the marc-pressed wines (P), the first dimension of the MFA contrasted the terms such as aggressive, pucker, and corduroy (positively projected on F1) with the subquality descriptors silk, velvet, soft, mouthcoat, full-body, and persistent (negatively projected on F1). Similarly, the BSA-reactive tannins and colour intensity were opposed to flavans and polymeric pigments. The MP and MS were equally correlated to F1, as their factor scores were −1.47 and −1.64, and highly differed from MS (1.2). The phenolic content and subquality variables contributed by 39.4% and 33% to the observations on F1. F2 correlated positively with the total anthocyanins, hue, and b* and negatively with the control, whose factor score was −1.66. The control wine was then associated with dry, adhesive, and green subqualities, which were mainly correlated with the factor F2.

In free-run wines, 50.8% of the experimental data variability was explained by F1, followed by 34.2% by F2 and 15% by F3 (Table 2). On F1, the BSA-reactive tannins, hue, polymeric pigments, and b* were correlated with the soft, rich, and full-body subqualities and the MS-treated wine. The colour parameters contributed mainly to this factor (43.2%). Conversely, F2 correlated with the total anthocyanin, colour intensity, aggressive, grainy, and pucker terms. The control was characterised by these variables, as shown by its factor score (−1.66). Opposite to the second factor, the MP correlated with the silk, velvet, and mouthcoat subqualities. Finally, MF was loaded on F3 and was mainly represented by subquality variables (53.3%), resulting in silk, persistent instead of dry, adhesive, green, and aggressive. Polymeric pigments also characterised this wine, positively correlated with F3 (0.691), although to a lesser extent than F1 (0.714).

### 2.5. The Effect of Mannoproteins on Aroma and Odour Descriptors

**Figure 3:**
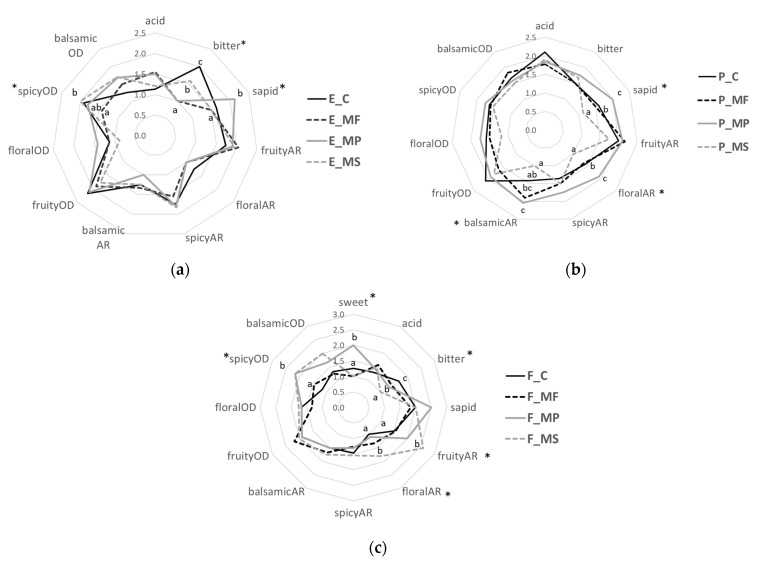
The spider charts representing the tastes, aromas (AR), and odours (OD) of (**a**) extended maceration (E), (**b**) marc-pressed (P), and (**c**) free-run (F) Sangiovese wines after six months of ageing. C represents the control wine. Mannoprotein treatments: MF, MP, and MS at 20 g/hL. The asterisk (*) indicates a statistical difference between wines with different letters according to Duncan’s test (*p* < 0.05).

In addition to mouthfeel, mannoproteins influenced the aroma and odour of the wines differently depending on the wine typology. In Figure 3a, the main effect of mannoproteins on taste was exerted in a wine with high flavan and tannin content (E).

The MP and MF were able to reduce the bitterness of extended macerated wine. Additionally, MP contributed to an increase in sapidity. The spicy odour was instead reduced in E-MF. For the marc-pressed wines (Figure 3b), a significant improvement was obtained by the MP mannoprotein. The P-MP revealed an increased floral and balsamic aroma and a high sapidity compared to other wines. In free-run wines (Figure 3c), mannoproteins modulated the flavour, increasing the fruity and floral aromas (F-MS), as well as the spicy odour (F-MS and F-MP). The three mannoproteins determined a reduction in bitterness, MS being the most effective. However, an increase in the sweetness perception was felt only in F-MP wine.

## 3. Discussion

Mannoproteins represent a natural oenological product that aims to improve the sensory characteristics of red wine, such as mouthfeel, astringency, bitterness, and colour. Several works have shown that treatments with mannoproteins increase the perception of sweetness and roundness sensation, body, persistence, aroma intensity, and odour complexity and reduces the astringency, bitterness, and aggressive green tannins [19,22,23,24]. In this work, we used Sangiovese wines with high tannin contents obtained by different winemaking processes, such as extended maceration and marc-pressing. After six months of ageing, significant differences were observed, with mannoproteins showing distinct behaviours according to wine typology. After this contact period, the MP precipitated vanillin-reactive flavans and BSA-reactive tannins, probably due to its high content in peptides. Peptides, having a high affinity towards high and low molecular weight proanthocyanidins, induce them to precipitate, reducing the final concentration in the treated wine. This result is more evident when the wine is richer in these compounds (E > P > F). A decrease in bitterness and an increase in sapidity in E-MP was also detected and could be due to the masking effect of the sapid peptide, which is a part of the formulation of MP. This peptide (Hsp12p), belonging to the heat shock proteins family, exhibited a sweet taste [25], thus conferring sapidity and reducing wine bitterness [16]. Furthermore, the treatment with MP seemed the most suitable for extended maceration wines, as it improved the wine’s mouthfeel by granting mouthcoating and soft and velvety sensations. This result is in accordance with previous work on Sangiovese, a wine rich in tannins and flavans, in which the MP was able to enhance the body, structure, and roundness of the wine [16].

From the multivariate analysis, the content of phenolic compounds in the treated wines was found to be differently correlated with the astringency subqualities and colour parameters according to the wine typology and mannoprotein treatment. In particular, it has been shown that the ratio of anthocyanins/tannins (A/T) in wines affects the formation of polymeric pigments during ageing [26]. When the wine has a high phenolic content (total anthocyanins, BSA-reactive tannins, and vanillin-reactive flavans) and the tannins are in excess with respect to anthocyanins (A/T = 0.2), as in the case of extended maceration wine, the decrease of phenolic compounds observed after six months of ageing with MP leads to a decreased astringency, felt as a dryness, hardness and unripeness. It equally resulted in the development of positive subqualities (velvet, soft, mouthcoat, satin, and persistent). Previous studies also showed that the addition of mannoproteins significantly modifies the mouthfeel and structural properties of red wines, leading to a reduction in astringency [22,27]. The decrease in phenolic content showed a more significant influence on the subqualities than on the formation of polymeric pigments. However, the colour stability of extended maceration wines was promoted by mannoproteins, which may allow multiple interactions between proanthocyanidins and anthocyanins, as observed by others [24,27].

In marc-pressed wines, where the content of proanthocyanidins was also in excess compared to anthocyanins (A/T ratio = 0.3), the high tannin content again correlated with negative astringency subqualities (dry, aggressive, hard, and pucker), as also reported for pressed wine fractions [11]. The higher the decrease in tannins detected after ageing in contact with MP, the more positive subqualities such as velvet, soft, mouthcoat, and full-body were obtained by the applied treatments (MP and MF). Moreover, the improvement in mouthfeel was correlated with polymeric pigment formation. Regarding the latter compounds, the sensory perception of the polymeric pigments such as velvety and mouthcoating was also observed during ageing [28]. Although the MF and MP mannoproteins similarly affected the wines, they differed more in their effects on colour and aroma than on mouthfeel. Specially, MF favoured more the formation or a smaller loss of polymeric pigments during ageing and then colour stability. In contrast, MP influenced the aroma revelation (more floral and balsamic) and sapidity.

In the free-run wines, the total anthocyanins accounted for half of the BSA-reactive tannin content and A/T = 0.5. Unlike the other wine typologies (P and E wines showed A/T < 0.5), the tannins were correlated with positive subqualities. The mannoproteins probably had some protective effects on the precipitation and depolymerisation of tannins when more anthocyanins were present, promoting the formation of stable macrostructures, which are less reactive towards salivary proteins and less astringent [29]. It is likely that BSA-reactive tannins remain in solution because: (i) the complexes between polymeric tannins and anthocyanins are stable [30], and (ii) mannoproteins contribute to the further stabilization of the complexes [31]. These hypotheses can be supported by the fact that the formation of polymeric pigments resistant to the action of SO_2_ was observed after all mannoprotein treatments. Concurrently, an improvement in mouthfeel was observed with MS > MF > MP. A reduction in bitterness was also observed in treated wines. An effect of polysaccharides on the bitterness was also previously reported [17,23]. From the MFA, a significant correlation between the positive subqualities, polymeric pigments formation, and decrease of flavans was found. This means that condensed flavans, when A/T = 0.5, are principally involved with mannoproteins in the formation of polymeric pigments, which are also characterised by an improved mouthfeel. Alcalde-Eon et al. [32] already proposed an additional mechanism that can explain these data, in which the steric hindrance caused by mannoproteins can protect the flavanol from precipitation and stabilise the interaction with anthocyanins. Ultimately, mannoproteins can improve the aroma of wine [17,33,34], because free-run MS-treated wine was perceived as more floral, fruity, and spicy than the control. Mechanisms involving orthonasal perceptions could explain this result.

## 4. Materials and Methods

### 4.1. Wine Samples 

Sangiovese wines were industrially produced in a winery located in the Chianti DOCG area (Toscana, Italy) during the 2016 vintage. Vinification was based on the following protocol: grapes (18 tons) were destemmed and crushed, the resulting must treated with potassium metabisulfite (40 mg/kg) and inoculated with 20 g/hL of yeast (F83 Laffort, Bordeaux, France); the fermentation/maceration lasted 12 days at 25 °C, during which yeast-assimilable nitrogen (YAN), in the form of diammonium phosphate (containing ≈0.12% of thiamine hydrochloride), was added with the inoculum and then again on the third and sixth days of fermentation, to a total concentration of 30 g/hL. The wine was then separated into three fractions: (i) extended maceration wine (E), which prolonged the skin contact for an additional 15 days, (ii) a devatted fraction of free-run wine (F), and (iii) a pressed fraction at 1.5 bar of marc-pressed wine (P). After completing the skin contact, E wine was pressed and, similarly to F and P, was transferred to 53-L carboys. After the addition of pectolytic enzymes (3 g/hL), the wines were inoculated with lactic bacteria (LF16 Direct, Laffort, Bordeaux, France) at 1 g/hL. Potassium metabisulfite (6 g/hL) was then added to the wines conserved under N_2_ in stainless-steel tanks (15 L) before commencing the experiments in October 2017.

### 4.2. Yeast Mannoprotein Products

Mannoproteins MF, MS, and MP were supplied by Laffort (Bordeaux, France). According to the manufacturer, MF is a specific yeast cell wall mannoprotein from *Saccharomyces cerevisiae* used for the colloidal stabilisation of wine and to improve the mouthfeel. MS is a specific mannoprotein (MP40—Patent 2726284) naturally present in wines and used to inhibit potassium bitartrate crystallisation. MP is a yeast cell wall extract composed of mannoproteins rich in a sweet peptide fraction (Patent EP 1850682) and vegetal polysaccharides (gum arabic). These products are qualified for use in oenology and comply with Regulation (EC) n° 606/2009.

Before ageing (t0), the E, P, and F wines were treated with MF, MP, and MS at 20 g/hL in duplicate. We used the concentration of 20 g/hL as the average dose recommended by the manufacturer (10–30 g/hL) to compare the products at the same concentration. The base parameters of the wines at t0 are shown in Supplementary Table S2. The control wine (C) was not treated. Two independent bottles (750 mL) were considered for each treatment and were stored in a cellar for six months. After this period, the wines were filtered under vacuum with Whatman^®^ glass microfiber filters (64 g/m^2^) (GE Healthcare, Chicago, IL, USA) before analysis.

### 4.3. Wine Sensory Evaluation

Sangiovese wines were evaluated in duplicate by 13 trained assessors (comprising five women between the ages of 35–50 and eight men between the ages of 25–44 years), as previously described [9,28]. Two tasting evaluations of four anonymous samples were conducted on each session. They were presented in balanced random order at room temperature (18 ± 2 °C) in black tulip-shaped glasses coded with 3-digit random numbers. The assessors were instructed to pour the whole sample in their mouth, hold it for 8 s, expectorate, and answer a check-all-that-apply (CATA) question with 16 sensory attributes of astringency. The attributes were the following: silk, velvet, dry, corduroy, adhesive, aggressive, hard, soft, mouthcoat, rich, full-body, green, grainy, satin, pucker, and persistent, defined in Supplementary Table S1. Judges waited for 4 min before rinsing twice for 10 s with mineral water (Sorgesana, pH ≈ 7) and then waited at least 30 s before drinking the following sample. The serving order design was a juxtaposition of Latin squares balanced for carryover effects [35]. The panel also evaluated the taste (sweet, acid, bitter, sapid); odour; and aroma (floral, fruity, spicy, balsamic) of the wines using a 5-point scale.

### 4.4. Chemical Analyses

All spectrophotometric determinations were performed using a Spectrophotometer Shimadzu UV-1800 model. Wine colour intensity (CI), given by the sum of the absorbances at 420, 520, and 620 nm and hue (420/520 Abs) were analysed using the Glories method [36]. CIELab allows the specification of colour perception in terms of a three-dimensional space. The L*-axis is known as the lightness and ranges from 0 (black) to 100 (white). The other two coordinates a* and b* represent redness–greenness and yellowness–blueness, respectively. CIELab coordinates were determined by Panorama software (Shimadzu, Milan, Italy). The total colour difference (ΔE) between two samples (treated wine and control) was obtained using the following expression: ΔE = [(ΔL*)^2^ + (Δa*)^2^ + (Δb*)^2^]^1/2^, in CIELab units [37]. Vanillin-reactive flavans were determined according to Di Stefano and Guidoni [38]. Total anthocyanins, polymeric pigments (LPP + SPP) as a measure of the colour stability of the wine, and BSA-reactive tannins were determined by the Harbertson et al. method [39]. Briefly, in this method, pH changes allow the evaluation of polymeric pigments by combining the analysis of the supernatant obtained after protein precipitation using bovine serum albumin (Sigma, Merck Life Science, Milano, Italy) for the tannin analysis (BSA-reactive tannins) and the bisulfite bleaching of the pigments in wine. For the determination of the total anthocyanins, 500 mL of wine diluted in a buffer solution (5-g/L potassium bitartrate,12% EtOH, and pH adjusted to 3.3 with HCl) were added to 1 mL of a buffer solution (200-mM maleic acid, 170-mM NaCl, and pH adjusted to 1.8 with NaOH) and incubated for 5 min. Total anthocyanins were determined by reading the absorbance of this solution at 520 nm. All analyses were carried out in duplicate on each bottle, for a total of four replicates.

### 4.5. Data Analysis

As a one-way ANOVA analysis, Fisher’s Least Significant Differences (LSD) procedure was used to distinguish the means of the phenolic and colour variables over four replicates. Sensory attributes (taste, odour, and aroma) were evaluated using Duncan’s test. Differences of *p* < 0.05 were considered significant. CATA responses were elaborated by the CATA analysis for each wine typology, and the most significant astringency subqualities (*p* < 0.01) were projected as explanatory variables in the CATA plot. A Multiple Factor Analysis (MFA) was performed on the frequency table containing responses to the CATA question, the phenolic content, and the colour parameters of the wines to investigate the relationships between the data from the chemical analyses and responses to the CATA question as separate groups of variables. Elaborations were carried out by means of XLSTAT software (Addinsoft, XLSTAT 2021).

## 5. Conclusions

Depending on the A/T ratio, each Sangiovese wine could necessitate a specific mannoprotein to improve the mouthfeel and/or colour. If there is a strong excess in tannins (extended maceration wines with A/T = 0.2), ageing with MP at 20 g/hL can be preferred, because it confers positive subqualities and colour stability to the wine. When A/T = 0.3, as in marc-pressed wines, both MF and MP can improve the mouthfeel and colour of Sangiovese. However, in free-run wine, where the A/T ratio is 0.5, the polymeric pigment formation was enabled by all treatments and correlated with an improved mouthfeel sensation. Further experiments on the role of the A/T ratio will be carried out to better understand the mechanisms involved in these phenomena. In addition, the bitterness was reduced by mannoproteins. For free-run wines, the ideal treatment can be represented by the MS, as it also showed a significant effect on the aroma revelation. In all cases, the formation or preservation of polymeric pigments by mannoproteins during ageing can be associated with positive subqualities, like velvet, soft, and mouthcoat.

## Figures and Tables

**Table 1:** The colour parameters of Sangiovese wines (E = extended maceration, P = marc-pressed, and F = free-run) aged on mannoproteins MF, MP, and MS for six months.

Wine Typology	Samples	Total Anthocyanins mg/L	Colour Intensity (420 + 520 + 620) a.u. ^†^	Hue	Polymeric Pigments (520) a.u. ^†^	L*	a*	b*	ΔE
Extended Maceration (E)	E-C	256.99 ± 0.84 a	13.70 ± 0.02 a	0.75 ± 0.00 b	5.87 ± 0.07 c	70.2 ± 0.2 c	37.6 ± 0.3 a	19.7 ± 0.3 a	-
	E-MF	252.13 ± 1.82 b	13.05 ± 0.02 c	0.75 ± 0.00 b	6.06 ± 0.09 b	71.5 ± 0.1 b	36.6 ± 0.0 b	19.3 ± 0.5 a	1.79 b
	E-MP	199.63 ± 0.44 d	11.98 ± 0.06 d	0.76 ± 0.01 a	6.08 ± 0.02 b	73.6 ± 0.1 a	34.1 ± 0.3 c	17.5 ± 0.6 b	5.34 a
	E-MS	228.97 ± 0.51 c	13.21 ± 0.05 b	0.75 ± 0.01 b	6.28 ± 0.04 a	71.4 ± 0.4 b	37.1 ± 0.4 ab	19.2 ± 0.3 a	1.48 b
Marc-Pressed (P)	P-C	174.51 ± 2.86 c	11.06 ± 0.47 ab	0.72 ± 0.01 b	3.77 ± 0.01 b	68.2 ± 0.4 b	28.8 ± 0.1 a	11.2 ± 0.2 a	-
	P-MF	175.74 ± 0.28 bc	10.76 ± 0.23 b	0.72 ± 0.00 b	4.02 ± 0.01 a	66.7 ± 0.5 c	29.2 ± 0.2 a	11.2 ± 0.4 a	1.58 b
	P-MP	178.92 ± 2.30 b	10.57 ± 0.16 b	0.73 ± 0.00 b	3.97 ± 0.09 ab	70.2 ± 0.4 a	26.8 ± 0.5 b	10.4 ± 0.3 b	2.98 a
	P-MS	192.03 ± 1.57 a	11.46 ± 0.13 a	0.75 ± 0.01 a	3.89 ± 0.20 ab	66.7 ± 0.2 c	29.0 ± 0.2 a	10.9 ± 0.5 a	1.58 b
Free-Run (F)	F-C	223.97 ± 4.98	12.63 ± 0.01 b	0.68 ± 0.00 b	4.42 ± 0.06 d	62.6 ± 0.3 b	49.3 ± 0.1 b	25.8 ± 0.1 c	-
	F-MF	219.71 ± 19.70	13.10 ± 0.26 a	0.70 ± 0.01 a	4.70 ± 0.05 b	62.8 ± 0.0 ab	49.7 ± 0.0 a	25.7 ± 0.1 c	0.52 b
	F-MP	207.78 ± 3.26	11.86 ± 0.01 c	0.69 ± 0.00 ab	4.54 ± 0.01 c	63.1 ± 0.0 a	49.4 ± 0.0 b	26.6 ± 0.1 b	0.91 b
	F-MS	218.82 ± 6.96	12.88 ± 0.07 ab	0.70 ± 0.00 a	4.80 ± 0.06 a	61.9 ± 0.2 c	48.1 ± 0.2 c	27.4 ± 0.2 a	2.11 a

**^†^** a.u. = absorbance unit. According to Fisher’s LSD analysis, values ± standard deviation (SD) with different letters indicate significant differences for each wine typology (*p* < 0.05).

**Table 2:** Relationships from the Multiple Factor Analysis between the variables: astringency subqualities (silk, velvet, dry, corduroy, adhesive, aggressive, hard, soft, mouthcoat, rich, full-body, green, grainy, satin, pucker, and persistent); colour parameters (colour intensity, hue, a*, b*, L*, and polymeric pigments); and phenolic content of the wine (total anthocyanins, BSA-reactive tannins, and vanillin-reactive flavans) and factors: C = control and mannoprotein treatments = MF, MP, and MS for extended maceration (E), marc-pressed (P), and free-run (F) wines.

	Extended Maceration Wines (E)			Marc-Pressed Wines (P)			Free-Run Wines (F)		
	F1	F2	F3	F1	F2	F3	F1	F2	F3
Eigenvalue	2.9	0.7	0.3	2.5	1.4	0.2	2.2	1.5	0.7
Variability (%)	75.9	17.5	6.6	61.2	35.0	3.7	50.8	34.2	15.0
Cumulative %	75.9	93.4	100.0	61.2	96.3	100.0	50.8	85.0	100.0
**Correlations**									
*Subquality*									
Silk	0.270	0.928	0.258	−0.838	0.354	0.416	0.132	−0.828	0.545
Velvet	0.984	−0.178	−0.010	−0.962	0.220	0.161	0.493	−0.868	0.054
Dry	−0.860	−0.471	−0.197	0.710	−0.703	0.051	0.101	0.623	−0.776
Corduroy	−0.064	0.834	−0.548	0.942	0.330	−0.058	−0.713	0.695	0.092
Adhesive	−0.942	−0.136	−0.308	0.464	−0.883	0.077	0.525	0.087	−0.846
Hard	−0.753	−0.600	−0.269	0.772	−0.495	0.398	−0.367	0.819	−0.441
Aggressive	−0.996	−0.088	0.013	0.867	−0.444	−0.226	−0.140	0.990	−0.033
Soft	0.917	−0.398	0.009	−0.823	0.551	−0.138	0.795	−0.587	−0.153
Mouthcoat	0.991	−0.097	−0.097	−0.869	0.491	0.060	0.143	−0.919	−0.368
Rich	0.439	0.389	0.810	−0.280	0.785	0.552	0.959	−0.192	−0.206
Green	−0.847	−0.488	−0.210	0.626	−0.776	0.076	−0.156	0.456	−0.876
Grainy	−0.948	0.102	0.302	0.765	−0.428	−0.482	−0.686	0.727	−0.038
Satin	0.970	−0.245	0.001	−0.393	0.795	−0.462	−0.994	−0.055	0.096
Pucker	−0.977	−0.207	−0.055	0.976	0.211	−0.052	−0.450	0.879	−0.157
Full-body	0.365	0.812	0.455	−0.826	0.506	−0.250	0.679	−0.342	0.650
Persistent	0.842	0.162	0.514	−0.993	0.098	−0.058	−0.341	−0.314	0.886
*Colour parameter*									
L*	0.997	−0.072	0.028	−0.776	−0.522	0.353	−0.853	−0.507	−0.122
a*	−0.957	0.287	−0.051	−0.752	−0.647	−0.127	−0.959	−0.191	0.210
b*	−0.961	0.232	0.147	0.378	0.846	0.376	0.941	−0.270	−0.202
Hue	0.935	−0.330	0.128	0.304	0.952	−0.046	0.971	−0.134	0.199
Colour Intensity	−0.988	0.146	−0.060	0.899	0.348	−0.267	0.177	0.670	0.721
Polymeric pigments	0.486	0.862	−0.143	−0.854	0.483	−0.193	0.714	−0.118	0.691
*Phenolic content*									
Total Anthocyanins	−0.930	0.002	0.368	0.454	0.889	0.066	−0.031	0.979	0.204
BSA-reactive tannins	−0.923	−0.342	0.176	0.976	−0.179	0.121	0.952	0.296	−0.076
Vanillin-reactive flavans	−0.908	0.373	−0.192	−0.962	0.269	0.038	−0.913	−0.370	0.169
**Factor scores**									
C	**−2.006**	−0.729	−0.215	1.486	**−1.660**	0.038	−1.106	**1.611**	−0.836
MF	−0.106	0.284	**0.897**	**−1.641**	−0.080	−0.523	−1.087	0.048	**1.260**
MP	**2.768**	−0.702	−0.169	**−1.467**	0.242	0.573	−0.469	**−1.882**	−0.615
MS	0.016	**1.248**	−0.429	**1.769**	1.763	−0.073	**2.465**	0.317	0.097
**Contributions (%)**									
Subquality	32.6	55.2	63.6	33.0	26.7	33.4	20.3	49.6	53.3
Colour parameter	33.7	30.1	5.2	27.6	43.7	60.3	43.2	13.5	41.3
Phenolic content	33.7	14.7	31.2	39.4	29.5	6.3	36.5	36.8	5.4

## Data Availability

Data are available upon request.

## Supplementary Materials

The following is available online. Table S1: Definitions of the attributes used to characterise the mouthfeel of wines. Table S2: Analyses of the base parameters of extended maceration (E), marc-pressed (P), and free-run (F) Sangiovese wines before ageing (t0).

## Sample Availability

Samples of the mannoproteins are available from the authors.
