# Supplementary material for: Commercial Mannoproteins Improve the Mouthfeel and Colour of Wines Obtained by Excessive Tannin Extraction
Source: Molecules. 2021 Jul 7;26(14):4133. doi: 10.3390/molecules26144133 (PMC8303419; doi:10.3390/molecules26144133)
Supplement: Supplementary file 1 [file molecules-26-04133-s001.zip › molecules-1254257-supplementary.pdf]

Table S1. Definitions of the attributes used to characterise the mouthfeel of wines.

Attribute	Definition
Silk	Tactile sensation like silk
Velvet	Tactile sensation like velvet
Dry	A feeling of lack of lubrication in the mouth
Corduroy	A sensation of a slight wrinkling of the soft palate that tongue movements can feel
Adhesive	The feeling that mouth surfaces are sticking yet can be pulled away from each other with slight pressure
Hard	Effect of astringency and bitterness
Aggressive	Excessive astringency of strong roughing nature
Soft	Light and finely textured astringency
Mouthcoat	Like a coating film that adheres to mouth surfaces
Rich	High flavour concentration with balanced astringency
Green	Combined effect of an excess of acidity and astringency
Grainy	A sensation of micro-particles in the mouth
Satin	A smooth and sliding astringency
Pucker	The reflex action of mouth surfaces being brought together and released in an attempt to lubricate mouth surfaces
Full-Body	A sensation of high viscosity
Persistent	An overall sensation (flavour, tactile, taste) which lasts over time

Table S2. Analyses of base parameters of extended maceration (E), marc-pressed (P), and free-run (F) Sangiovese wines before aging (t0).

	Alcohol (% v/v)	pH	Tritatable acidity (g/L tartaric acid)	Volatile acidity (g/L acetic acid)	free SO_2 (mg/L)	total SO_2 (mg/L)
E-t0	14.20 ± 0.47	3.17 ± 0.01	6.7 ± 0.4	0.5 ± 0.0	19.1 ± 0.2	33.3 ± 0.9
P-t0	13.02 ± 0.04	3.31 ± 0.01	6.2 ± 0.0	0.5 ± 0.0	24.2 ± 2.4	35.1 ± 3.2
F-t0	13.17 ± 0.11	3.24 ± 0.00	6.3 ± 0.2	0.4 ± 0.0	22.1 ± 1.4	35.2 ± 2.3
